# Architectural properties of the musculoskeletal system in the shoulder of two callitrichid primate species derived from virtual dissection

**DOI:** 10.1007/s10329-021-00917-7

**Published:** 2021-06-28

**Authors:** Lennart Eigen, John A. Nyakatura

**Affiliations:** Comparative Zoology, Institute of Biology, Humboldt University of Berlin, Philippstraße 13, 10115 Berlin, Germany

**Keywords:** ACSA, Muscle architecture, Muscle moment arm, Muscle maps, *Callitrichidae*

## Abstract

Callitrichidae are small, arboreal New World primates that utilize a variety of locomotor behaviors including trunk-to-trunk leaping (TTL) and horizontal locomotion which involve differential functional demands. Little is known about the relationship between the preferred locomotor behavior and musculoskeletal architecture of these primates. In this study, we compared the musculoskeletal architecture of selected shoulder muscles in two cadavers each of the trunk-to-trunk leaper *Cebuella pygmaea* and the mainly pronograde quadrupedally moving *Saguinus imperator subgrisescens*. Contrast-enhanced microfocus computed tomography (µCT) was used to virtually dissect the cadavers, produce muscle maps, and create 3D reconstructions for an image-based analysis of the muscles. Muscle lengths, muscle volumes, and osteological muscle moment arms were measured, and the anatomical cross-sectional areas (ACSA) were calculated. We expected the muscles of the forelimb of *S. imperator* to be larger in volume and to be relatively shorter with a larger ACSA due to a higher demand for powerful extension in the forelimbs of this horizontally locomoting species. For *C. pygmaea*, we expected relatively larger moment arms for the triceps brachii, supraspinatus, infraspinatus and subscapularis, as larger moment arms present an advantage for extensive vertical clinging on the trunk. The muscles of *S. imperator* were relatively larger in volume than in *C. pygmaea* and had a relatively larger ACSA. Thus, the shoulder muscles of *S. imperator* were suited to generate relatively larger forces than those of *C. pygmaea*. Contrary to our expectations, there were only slight differences between species in regard to muscle lengths and moment arms, which suggests that these properties are not dependent on the preferred locomotor mode. The study of this limited dataset demonstrates that some but not all properties of the musculoskeletal architecture reflect the preferred locomotor behavior in the two species of Callitrichidae examined.

## Introduction

The adaptation of an organism to different lifestyles is often reflected in its anatomy. Thus, investigating the anatomy quantitatively and qualitatively in a comparative framework can provide information about the adaptations of organisms to their respective environments. With the help of 3D visualization and digital analysis, advancements in imaging methods have made possible an image-based examination of the soft tissues that critically contribute to the musculoskeletal system (Metscher 2009a; Pauwels et al. 2013; Descamps et al. 2014). The musculoskeletal architecture has a significant influence on function (Lieber et al. 1992). This also includes the internal structure of a muscle as well as parameters that describe the effect of the muscles on the skeleton, such as the moment arms of a muscle to a joint. Forelimb osteological differences have been demonstrated to reflect differences in locomotor behavior (e.g., Fleagle and Meldrum 1988; Larson 2015; Wölfer et al. 2019). In addition to the osteological properties, limb function is obviously determined by muscle properties, such as force-generating capacity and contraction speed and distance, which are determined by muscle architecture (e.g., Gans and Bock 1965; Lieber et al. 1992; Lieber and Fridén 2000; Michilsens et al. 2009; Allen et al. 2010; Kikuchi 2010; Rosin and Nyakatura 2017; Marchi et al. 2018; Olson et al. 2018; Taverne et al. 2018; Nyakatura et al. 2019; Regnault et al. 2020).

### Muscle architectural properties

Studies of the variation in limb muscle anatomy in primates have shown that limb muscles of different species differ depending on the preferred locomotor behavior and body size (e.g., Demes et al. 1998; Leischner et al. 2018). Muscle architecture is defined as the three-dimensional arrangement of the muscle fibers within a muscle (Lieber et al. 1992). The architecture of a muscle is by and large consistent between individuals of the same species (Lieber and Fridén 2001). As a result, the quantification of the parameters of the muscles is crucial for the interpretation of anatomical adaptations and specializations.

There is a fundamental trade-off in the architecture of a muscle. For a given volume, muscles can be fast (a few long muscle fibers) or powerful (many short muscle fibers) (Zajac 1992; Allen et al. 2010; Kikuchi 2010; Rosin and Nyakatura 2017). In order for a muscle to generate more force, either the volume must be increased, or the muscle architecture must be changed accordingly (Allen et al. 2010). In muscles of the same volume, an “in-series” arrangement of fibers has a greater potential to contract, since the contraction potential is proportional to the number of contractile units along the line of action (Lieber and Fridén 2001; Allen et al. 2010). The length of the muscle fiber bundles, the fascicles, thus reflects the working area of a muscle (Rosin and Nyakatura 2017). They also have a greater potential to move faster, as each muscle fiber theoretically contracts at the same speed and potentially at the same time, and longer muscle fibers can thus span a greater distance in a given time (Zajac and Gordon 1989; Zajac 1992; Leischner et al. 2018). Force-generating capacity of a muscle is usually derived in anatomical studies from physiological cross-sectional area (PCSA), which has been shown to be a good indicator of force-generating capacity (e.g., Zajac and Gordon 1989; Zajac 1992; Kikuchi 2010; Rosin and Nyakatura 2017). It can be calculated from the pennation, i.e., the angle of the muscle fibers’ long axes from the muscle’s line of action, muscle mass, and muscle density (q) (1.06 g/cm^3^) (Méndez 1960; Kikuchi 2010). The PCSA provides a better prediction of the strength of a muscle than the volume (i.e., the mass) of a muscle (Payne et al. 2006; Leischner et al. 2018). Muscles with a large PCSA have a large number of fascicles arranged in parallel, resulting in a large number of fibers acting simultaneously to generate force. In the current study, we were not able to determine fascicle length and pennation angle from microfocus computed tomography (µCT) data; thus the anatomical cross-sectional area (ACSA) was quantified as an alternative. The ACSA sets the volume of a muscle in relation to its length without taking the pennation angle and fascicle length into account. As an indicator of force generation, it is therefore less accurate than the PCSA. Still, the larger the ACSA, the more likely this muscle is adapted to movements that require great force (Ikai and Fukunaga 1968). If a muscle is required to contract both powerfully and quickly over a longer distance, then it must have a larger volume than those that are only specialized for either larger or more powerful contractions (Anapol and Barry 1996).

### Muscle moment arms

**Fig. 1:**
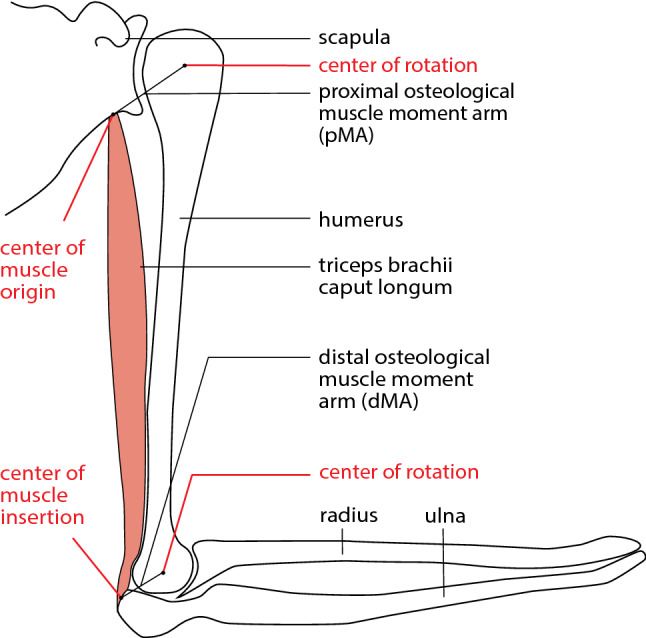
Schematic illustration of the distal (dMA) and proximal (pMA) osteological muscle moment arm (OMMA) exemplarily for triceps brachii caput longum

The moment arm converts the force generated by the muscle into a torque that causes the skeletal element to rotate around a joint. It thus often reflects its role, for example as a stabilizer or prime mover (Ackland et al. 2008). Comparative studies on moment arms, for example, succeeded in identifying specializations of the musculoskeletal system regarding locomotor or masticatory behaviors (e.g., Michilsens et al. 2009; Channon et al. 2010; Regnault et al. 2017). It was shown that the moment arm of the hindlimb muscles in gibbons are adapted to the locomotor demands. Their hip and knee extensors have larger muscles with shorter fascicles and relatively smaller moment arms. This functional adaptation enables rapid joint rotation with powerful movements that are required for certain locomotor tasks such as climbing and leaping (Channon et al. 2010). A distinction can be made between the instantaneous muscle moment arm and the osteological muscle moment arm (here abbreviated as OMMA) (Fig. 1). The instantaneous muscle moment arm is defined as the perpendicular distance from a muscle’s line of action to the skeletal element’s center of rotation (Cartmill et al. 1985; Ackland et al. 2008; Williams et al. 2008). The OMMA is a measure of the distance between the center of rotation of the joint and the attachment point of the muscle (Murray et al. 2002). In our analysis, we chose the OMMA because it does not depend on the instantaneous joint position. By using a 3D model based on a µCT scan, it is possible to measure the distance between muscle attachment points and the center of rotation directly.

###  Callitrichid primate locomotion

Callitrichidae belong to the New World monkeys. Their range extends from northern Bolivia to Panama (Buckner et al. 2015), and they differ, among other things, in their habitat use and preferred mode of locomotion (e.g., Garber and Pruetz 1995; Nadjafzadeh and Heymann 2008; Nyakatura and Heymann 2010). Studies of the locomotor behavior of diverse Callitrichidae show that species that inhabit different layers of a forest are specialized in different movement patterns (Garber and Pruetz 1995), which has led to the evolution of a differentiated musculoskeletal anatomy (Fleagle and Mittermeier 1980; Hunt et al. 1996). Two main modes of preferred leaping behavior have been described within the Callitrichidae (Prost 1965): horizontal leaping (HL) and vertical clinging and leaping, which is similar to trunk-to-trunk leaping (TTL) performed by *C. pygmaea*. Callitrichidae that specialize in HL usually move in a pronograde posture mainly on horizontal and oblique, often terminal branches and leap from these supports onto the next horizontal, terminal branch (Schmidt 2011). The locomotor behavior of most tamarins is largely characterized by HL as they move through the rainforest through a series of long, acrobatic jumps that usually start and end on very thin branches (Garber 1980). They mostly avoid vertical surfaces (Garber 1980). As a representative of this type of locomotion, *Saguinus imperator subgrisescens* (the bearded emperor tamarin, *S. imperator* (SIS) for brevity) is examined in this study. With an average body mass of 475 g, *S. imperator* belongs to the medium-sized New World Monkeys (Peres 1993; Smith and Jungers 1997). Horizontal leaping (32%) and walking (34%) are the dominant modes of locomotion in *S. imperator*. Other important behaviors but which are not used as frequently are claw climbing (12%) and bounding (8%) (Karantanis 2010). *Saguinus imperator* use oblique supports (50%) more often than horizontal supports (35%), and use vertical supports (15%) the least (Buchanan-Smith et al. 2000).

Trunk-to-trunk leaping is unusual for tamarins and is more likely to be observed in marmosets, especially in smaller representatives (e.g., Fleagle and Mittermeier 1980; Garber 1980, 1991; Garber and Sussman 1984; Stafford et al. 1994; Garber and Pruetz 1995; Youlatos 1999). In TTL, the monkeys leap from a vertical position from one trunk to another one and also lands in a vertical position (Youlatos 1999). This positional behavior can be observed in *Cebuella pygmaea* (pygmy marmoset), the second species examined here. *Cebuella pygmaea* (CP) is the smallest member of the Callitrichidae, with an average body mass of just 150 g, and field studies show that *C. pygmaea* uses claw climbing, clinging, and vertical surfaces more than any other representative of the Callitrichidae (Youlatos 2009). Nevertheless, a large repertoire of locomotor behavior can be observed in *C. pygmaea*, too, including horizontal walking and running as well as horizontal leaping between branches and other surfaces. Climbing (28%) was the most common behavior, followed by walking (23%) (Karantanis 2010). *Cebuella pygmaea* use vertical supports (55%) most of the time. Oblique supports (27%) are utilized less and horizontal supports (18%) the least (Buchanan-Smith et al. 2000). However, of all leaps, 85% have been documented to be from or to a vertical underground (Kinzey et al. 1975). The prevalence of locomotion on vertical supports is typical of *C. pygmaea*, since they feed more often on vertical supports and cling onto trunks while feeding on exudates (Youlatos 1999; Karantanis 2010).

### Expected differences in shoulder and brachial anatomy and aim of this study

The cadaveric samples of two species (a total of four specimens) were stained and then scanned with a µCT scanner. Three-dimensional reconstruction of shoulder and brachial muscles was used to assess muscle volume, muscle lengths, osteological muscle moment arms (OMMA), and anatomical cross-sectional area (ACSA) to test for expected anatomical differences that potentially reflect adaptations to differing functional demands induced by diverging locomotor behavior.

We examined the musculoskeletal system of a limited dataset of these two primates, which differ in their preferred mode of locomotion (e.g., Fleagle and Mittermeier 1980; Garber 1980, 1991; Youlatos 1999, 2009), using contrast-enhanced µCT. We expected that these differences in locomotor behavior are reflected in the anatomy of the two species. We focused on the intrinsic muscles that span the shoulder joint and muscles of the upper arm in our attempt to identify potential adaptations in the musculoskeletal system. Eight different muscles were examined (*n* = 32 muscles in total). These included m. biceps brachii, m. triceps brachii, m. brachialis, m. deltoideus, m. supraspinatus, m. infraspinatus, m. teres major, and m. subscapularis. The infraspinatus, supraspinatus, and subscapularis are part of the rotator cuff and important for the stabilization of the shoulder joint (Roberts 1974). Teres major is responsible for humeral retraction (Larson and Stern 1986, 2013). The supraspinatus assists the deltoideus in providing the force for elevation of the arm. The infraspinatus depresses the humeral head, ensuring that the humerus is not displaced but rather raised (Larson and Stern 1986). Biceps brachii and brachialis are elbow flexors; triceps brachii is an elbow extensor and a humerus retractor (Rupert et al. 2015). Elbow flexion and extension, and limb extension are important in arboreal locomotion, because to maintain balance, arboreal species need to rotate the forelimb forcefully (Larson and Stern 2006).

For *S. imperator* we expected relatively shorter muscles with a larger volume in relation to body size, since larger primates generally have relatively more powerful muscles in the forelimbs than smaller primates (Leischner et al. 2018). Since in HL the forelimbs can be expected to substantially contribute to body propulsion via humerus retraction (Schmitt 2003; Hesse et al. 2015), we expected relatively larger force-generating capacity as reflected in a relatively larger ACSA than in the TTL species. Primates such as *C. pygmaea*, that use TTL, are expected to have relatively long muscles with low volume in the forelimb compared to primates that prefer HL (Leischner et al. 2018). This might be an indicator of large muscle contraction distances and high contraction speeds which are important components of locomotion in a vertical clinging position (Huq et al. 2015; Leischner et al. 2018). We also expected relatively larger moment arms in the TTL species, which involves vertical clawing on tree trunks over a long period of time, and this holding should be reflected in an anatomy that favors torque generation without the need for the constant generation of large muscle forces.

## Materials and methods

One male and one female of each species *S. imperator* (SIS) and *C. pygmaea* (CP) were provided frozen and without internal organs by the Antwerp Zoo (Royal Zoological Society of Antwerp), where they lived in a group. The age of the animals is known (Table 1). After thawing the carcasses for one day, the head-torso length was measured, the body mass (excluding the inner organs) was determined, and finally they were skinned. The animals were fixed to kebab skewers with cable ties and then immersed in formaldehyde (ROTI-Histofix 4%; acid-free (pH7) phosphate-buffered formaldehyde solution 4%) for 9 or 17 days (Table 1). Next, specimens were immersed in successive alcohol baths of increasing concentration. The animals were treated as follows: 1 h in a water bath, 1 h in 15% ethanol, 1 h in 30% ethanol, 1 h in 50% ethanol, 1 h in 60% ethanol, and 1 h in 70% ethanol (Metscher 2009a, b; Pauwels et al. 2013; Descamps et al. 2014). The last step is staining with PTA (3% (w/v) phosphotungstic acid in 70% ethanol), which serves as the contrast medium for subsequent µCT scans (Koç et al. 2019).

**Table 1:** Samples and staining protocol of samples with phosphotungstic acid (PTA)

Species	*S. imperator*	*S. imperator*	*C pygmaea*	*C pygmaea*
Identification	M10981	M10924	M10029	M11128
Abbreviation	SIS1	SIS2	CP1	CP2
Head/trunk length (mm)	230	235	120	140
Length of hind limb (mm)	120	125	80	85
Length of front limb (mm)	70	85	70	72
Eviscerated body mass (g)	348	262	60	50
Age (years)	15	7	2	5
Sex	Female	Male	Female	Male
Staining				
Formaldehyde	9 days	2 days	17 days	2 days
0.3% PTA	18 days	–	18 days	–
3% PTA	15 days	15 days	15 days	15 days
3% PTA (refreshed)	14 days	14 days	14 days	14 days

The shoulders of *S. imperator* and *C. pygmaea* were scanned at the Museum für Naturkunde Berlin with a YXLON FF35 CT (YXLON International GmbH, Hamburg, Germany). A helix scan with a detector time of 0.8 s was carried out on all objects. For both specimens of *C. pygmaea*, 1886 image slices with a voxel size of 0.0324 × 0.0324 × 0.0324 mm made up the image stack. The scan parameters were 135 µA and 170 kV. For *S. imperator* 1965 images made up the complete stack. For SIS1 the voxel size is 0.0413× 0.0413 × 0.0413 mm; for SIS2 it is 0.0456 × 0.0456 × 0.0456 mm. The scan parameters were 135 µA and 180 kV.

**Fig. 2:**
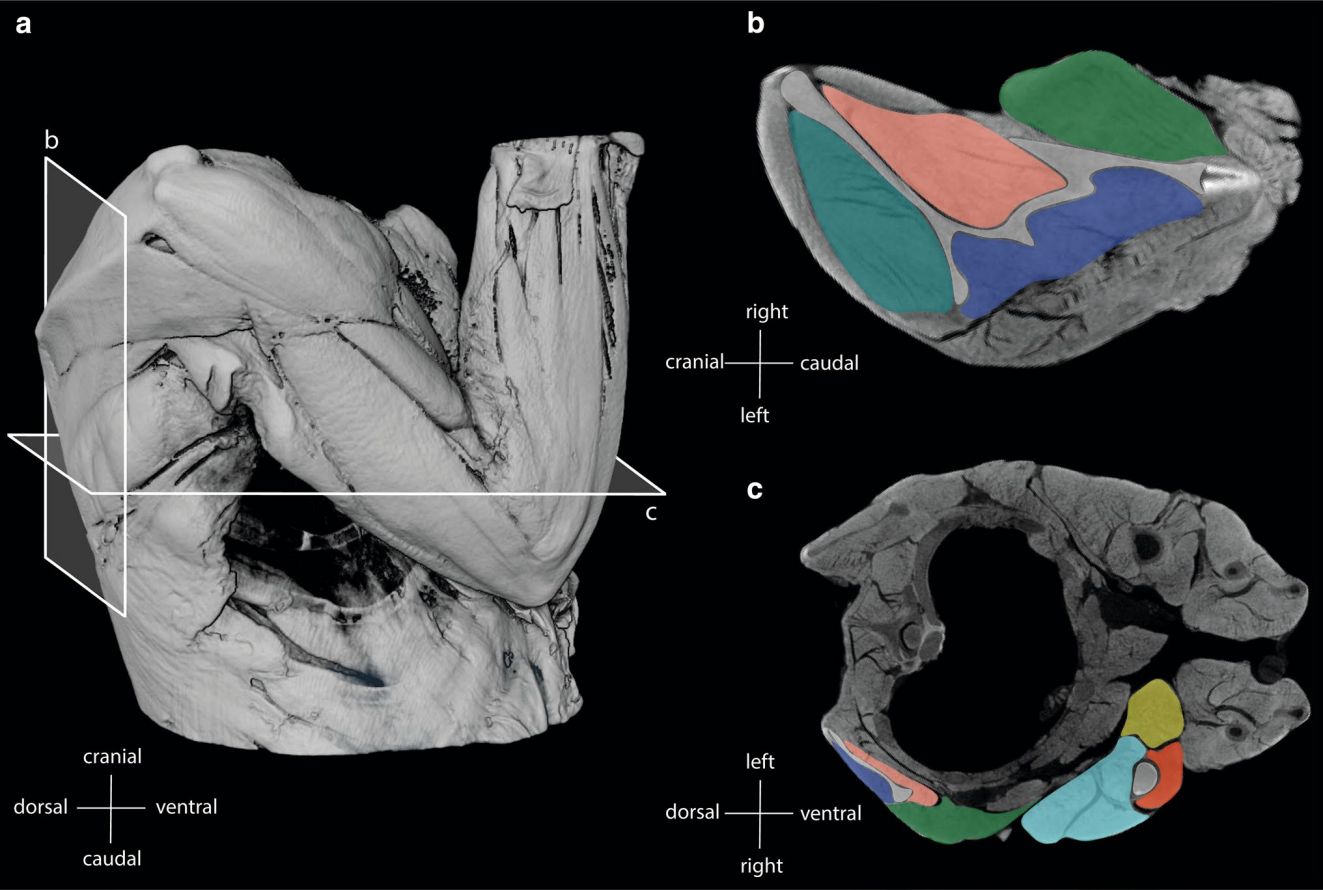
**a** Lateral view of the right shoulder of *S. imperator* (SIS1); **b** frontal plane cross-section as shown in **a**; **c** transverse plane cross-section as shown in **a**; biceps brachii: yellow; triceps brachii: turquoise; brachialis: red; supraspinatus: dark turquoise; infraspinatus: blue; teres major: dark green; subscapularis: pink

For the segmentation of muscles and bones and labeling as well as the calculation of the volumes, AMIRA software (version 6.0.0., Thermo Fisher Scientific) was used (Fig. 2). A 3D model was created, and the file was saved as an AVI file (.avii). These files were processed using ImageXd software (Heiko Stark, Jena, Germany; URL: http://starkrats.de), which enables the individual muscles and bones to be cut out and a mesh file (.mesh) to be created for each. The volume for each muscle was calculated automatically in AMIRA. The mesh files of the individual muscles were loaded into Autodesk Maya (Autodesk, Inc., San Rafael, CA, USA) and the length of the muscles and the moment arms were measured using the “distance measure” tool. First, the humerus of each individual was measured by dissection and then digitally in Autodesk Maya. This enabled a conversion factor to be determined for the Maya units obtained in Autodesk Maya, which was used for all muscles and skeletal elements. Next, we measured the muscle length from the center of origin to the center of insertion of the muscle. The distal OMMA (dMA) is the distance between the center of insertion of a muscle and the center of the joint on which the muscle acts. The proximal moment arm (pMA), on the other hand, is the distance between the center of the origin of a muscle and the center of the joint it acts on (Fig. 1).

After measuring the muscle lengths and the distal and the proximal OMMA, the skeletal element of each moment arm was measured. With the determined length and volume of the muscles, the ACSA was calculated using the following formula (Schumacher 1961):

$${\text{ACSA}}\; = \;{\text{Volume}}/{\text{Length}}$$

In addition to the absolute values, we compared the muscle volume, length, and the ACSA in relation to the average body mass of the corresponding species. To account for the differences in size, the muscle volume (muscle volume/body mass), muscle length (muscle length/body mass^0.33^), and ACSA (ACSA/body mass^0.66^) were standardized by average body mass under the assumption of isometric scaling (Allen et al. 2010). Moment arms are standardized by the length of the respective connecting skeletal element. The average mass for *S. imperator* is 475 g (Smith and Jungers 1997) and for *C. pygmaea* 150 g (Youlatos 2009).

## Results

### Muscle topology

**Fig. 3:**
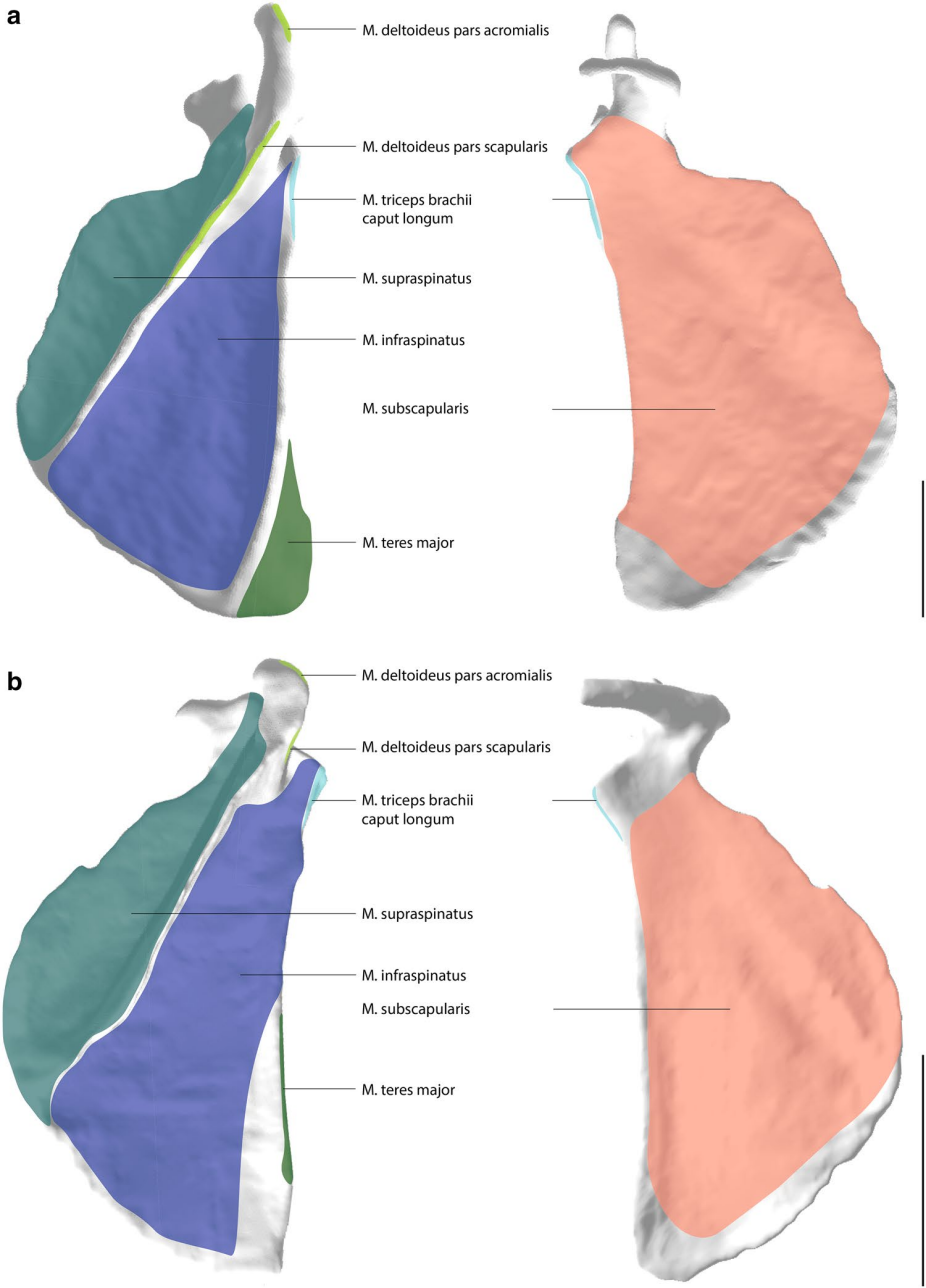
Muscle maps of the shoulder musculature attaching to the scapula. **a** *S. imperator* **b** *C. pygmaea;* left: lateral view of a right scapula; right: medial view of a right scapula. Scale bar = 10 mm

**Fig. 4:**
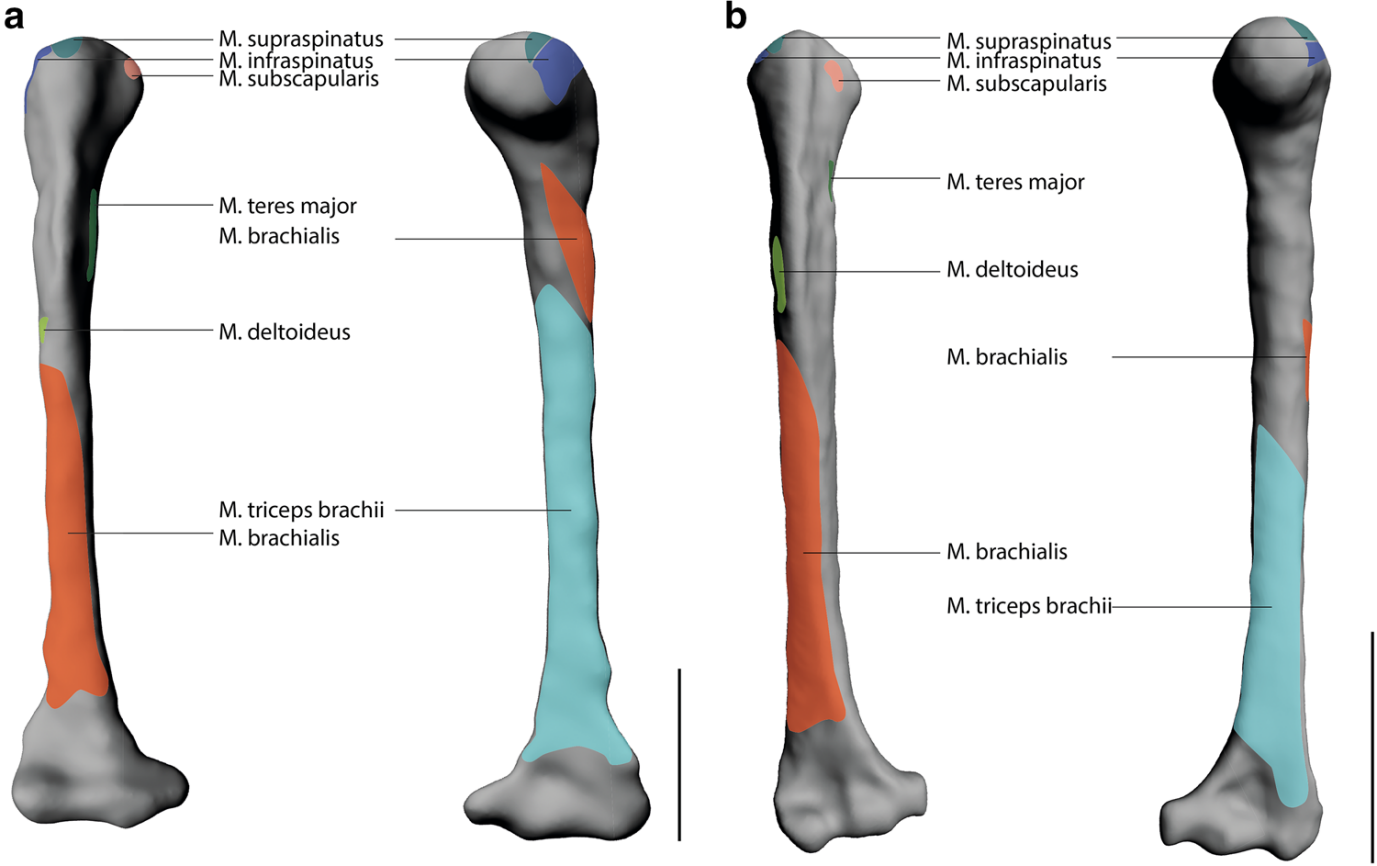
Muscle maps of the shoulder musculature attaching to the scapula. **a** *S. imperator* **b** *C. pygmaea;* left: lateral view of a right humerus; right: medial view of a right humerus. Scale bar = 10 mm

**Fig. 5:**
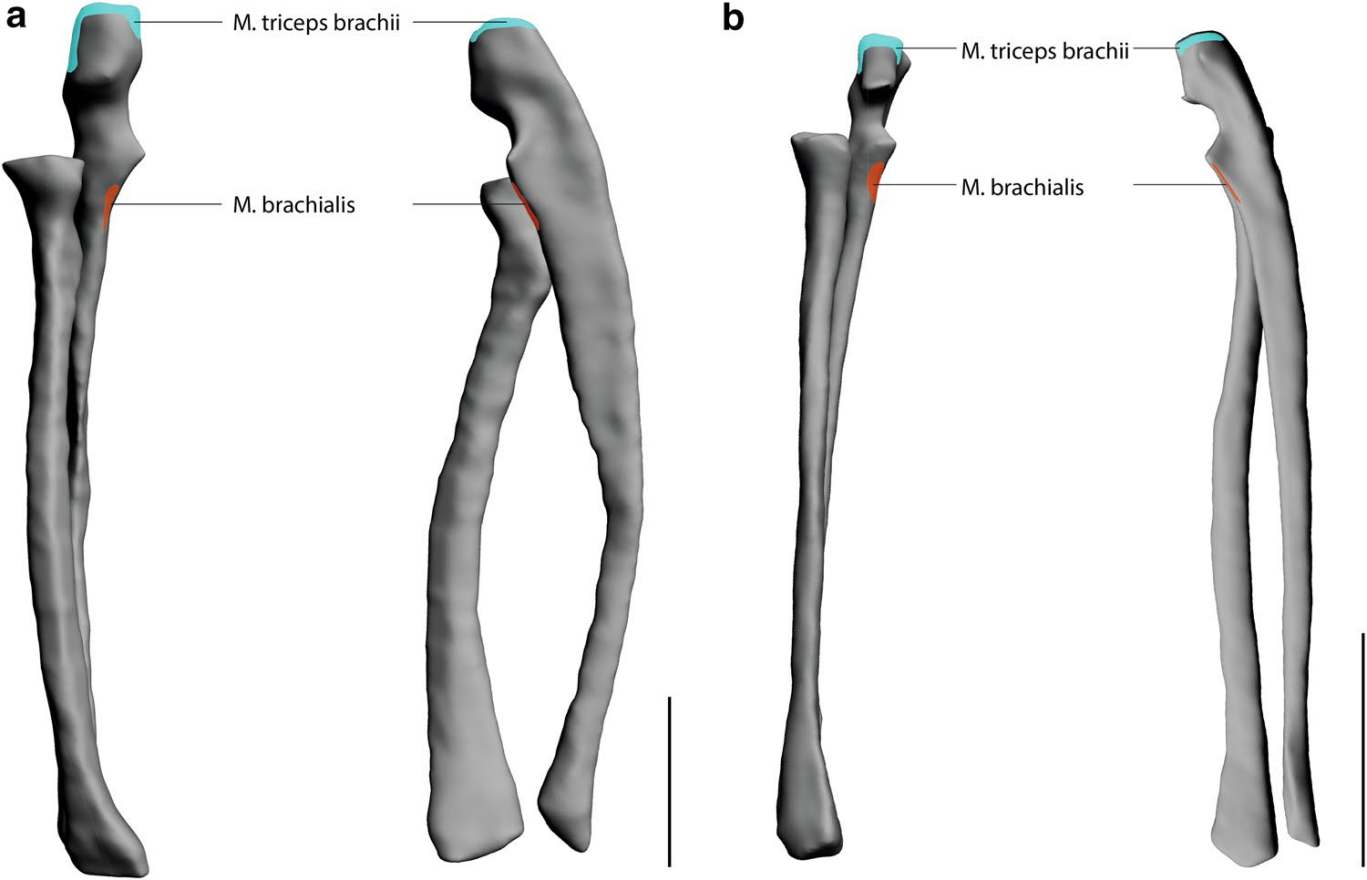
Muscle maps of the shoulder musculature attaching to the radius und ulna. **a** *S. imperator* **b** *C. pygmaea;* left: lateral view of a right radius and ulna; right: medial view of a right radius and ulna. Scale bar = 10 mm

**Fig. 6:**
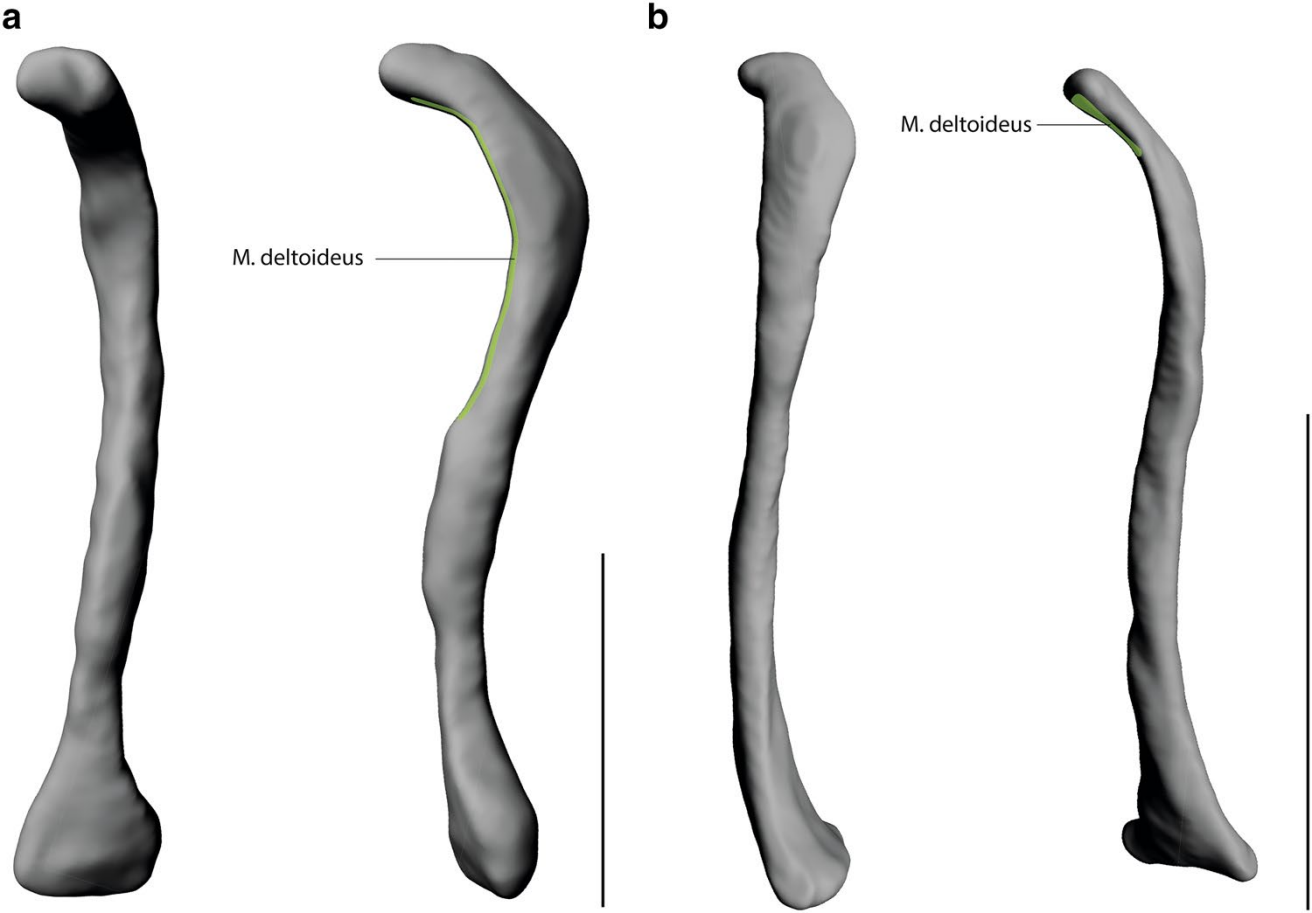
Muscle maps of the shoulder musculature attaching to the clavicula. **a** *S. imperator* **b** *C. pygmaea;* left: lateral view of a right clavicula; right: medial view of a right clavicula. Scale bar = 10 mm

Overall, the muscle attachment areas are similar in both species examined. However, there are also some differences. Biceps brachii caput longum originates at the tuberculum supraglenoidale. The origin of biceps brachii caput breve is at the processus coracoideus. The insertion of biceps brachii is at the tuberositas radii. The origin area of triceps brachii caput longum is at the tuberculum infraglenoidale and has approximately the same size in both species (Fig. 3). The origin area for triceps brachii caput mediale and laterale is at the facies posterior of the humerus and is relatively larger in *S. imperator* than in *C. pygmaea* (Fig. 4). The relative size of the insertion area for triceps brachii is at the olecranon and has approximately the same size in both species (Fig. 5). The brachialis originates at the facies anterior of the humerus, and the relative size of its attachment area is one and a half times larger in *S. imperator* than in *C. pygmaea* (Fig. 4). The insertion area for brachialis is at the tuberositas ulnae and has approximately the same relative size in both species (Fig. 5). Deltoideus pars acromialis originates at the acromium and the origin area is relatively the same size in both species. Deltoideus pars scapularis has its origin on the spina scapulae and the attachment area is three times larger in *S. imperator* than in *C. pygmaea* (Fig. 3). Deltoideus pars clavicularis has its origin in the acromial third of the clavicle. In *S. imperator*, the attachment area of this muscle is three times larger than in *C. pygmaea* (Fig. 6). The insertion of deltoideus in both species is on the tuberositas deltoidea (Fig. 4). The teres major originates at the margo lateralis and the attachment area is relatively larger in *S. imperator* compared to *C. pygmaea* (Fig. 3). The insertion of teres major is at the crista tuberculi and relatively smaller in *C. pygmaea* compared to *S. imperator* (Fig. 4). The origin of infraspinatus is at the fossa infraspinata and the attachment area is relatively slightly larger in *S. imperator* than in *C. pygmaea* (Fig. 3). The insertion area of infraspinatus is at the caput humeri and relatively larger in *S. imperator* (Fig. 4). The supraspinatus originates at the fossa supraspinata and the attachment area is relatively the same size in both species (Fig. 3). The insertion area of supraspinatus is at the caput humerus and has approximately the same relative size in both species (Fig. 4). The subscapularis originates at the fossa scapularis and the relative size of the attachment area is the same in both species (Fig. 3). The insertion area is at the tuberculum minus and has relatively the same size in both species (Fig. 4).

### Muscle volume

**Fig. 7:**
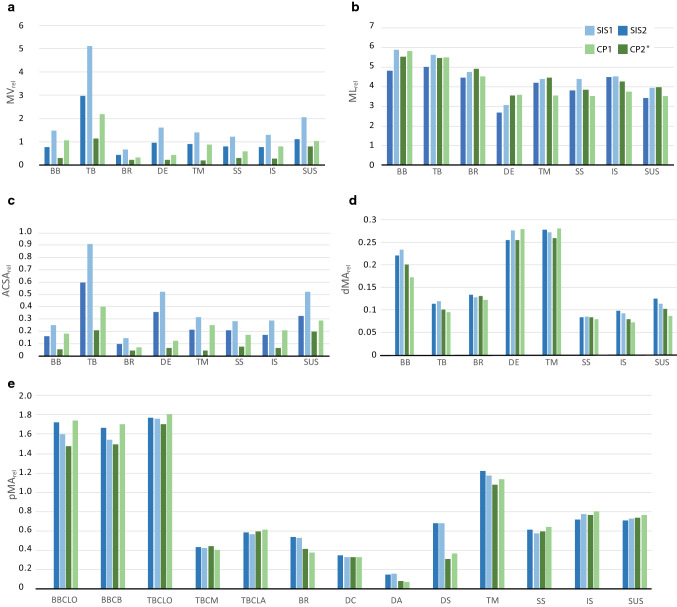
Muscle architectural parameters of *S. imperator* and *C. pygmaea* **a** relative muscle volume (MV_rel_), **b** relative muscle length (ML_rel_), **c** relative ACSA (ACSA_rel_), **d** distal relative moment arms (dMA_rel_), **e** proximal relative moment arms (pMA_rel_). BB: m. biceps brachii; BBCLO: biceps brachii caput longum; BBCB: biceps brachii caput breve; TB: m. triceps brachii; TBCLO: triceps brachii caput longum; TBCM: triceps brachii caput mediale; TBCLA: triceps brachii caput laterale; BR: brachialis; DE: deltoideus; DC: deltoideus pars clavicularis; DA: deltoideus pars acromialis; DS: deltoideus pars spinalis TM: teres major; SS: supraspinatus; IS: infraspinatus; SUS: subscapularis

**Fig. 8:**
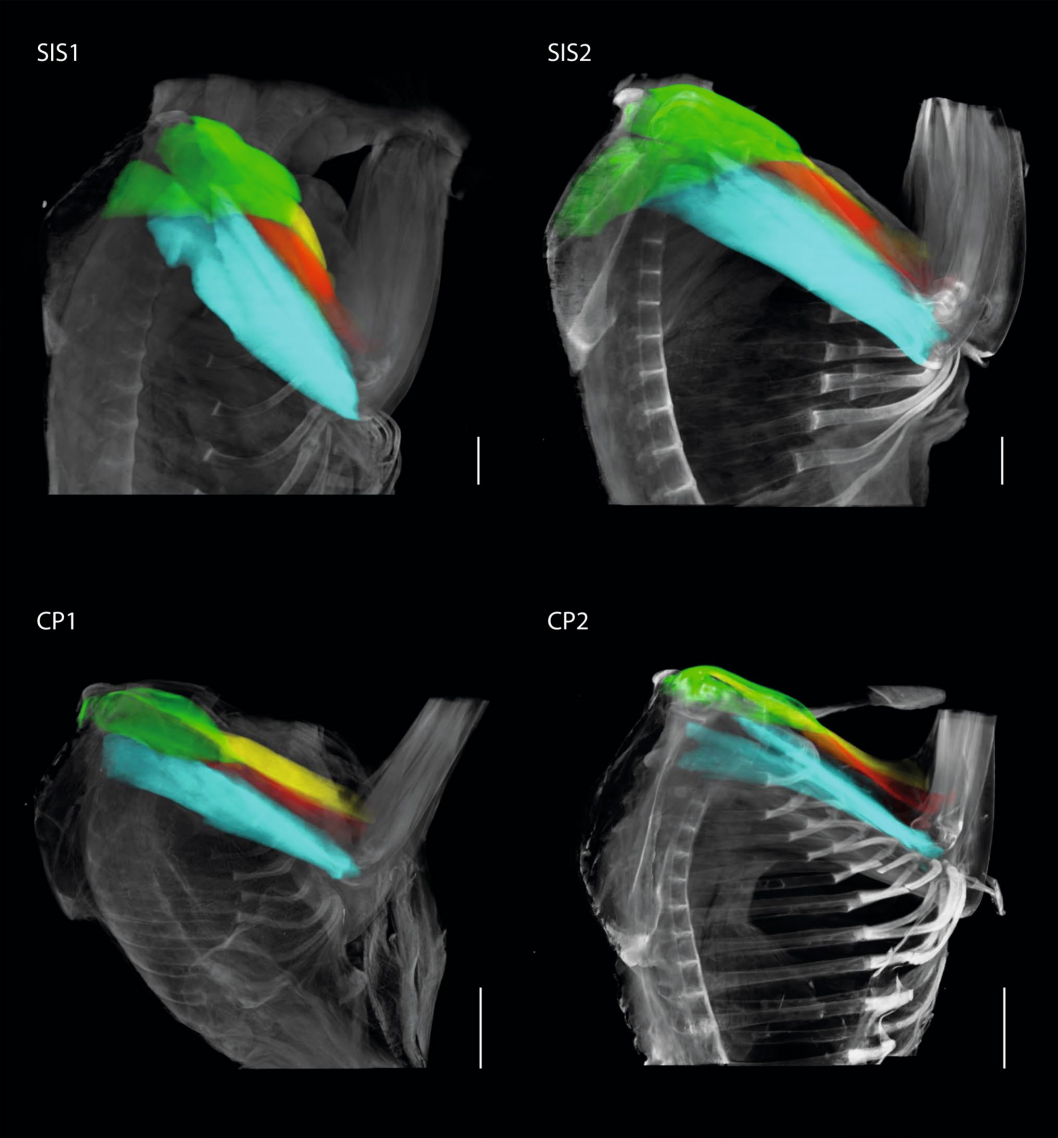
Lateral view of the upper arm muscles of *S. imperator* and *C. pygmaea.* SIS1 and SIS2: *S. imperator;* CP1 and CP2: *C. pygmaea;* biceps brachii: yellow; triceps brachii: turquoise; brachialis: red; deltoideus: light green. Scale bar = 10 mm

**Fig. 9:**
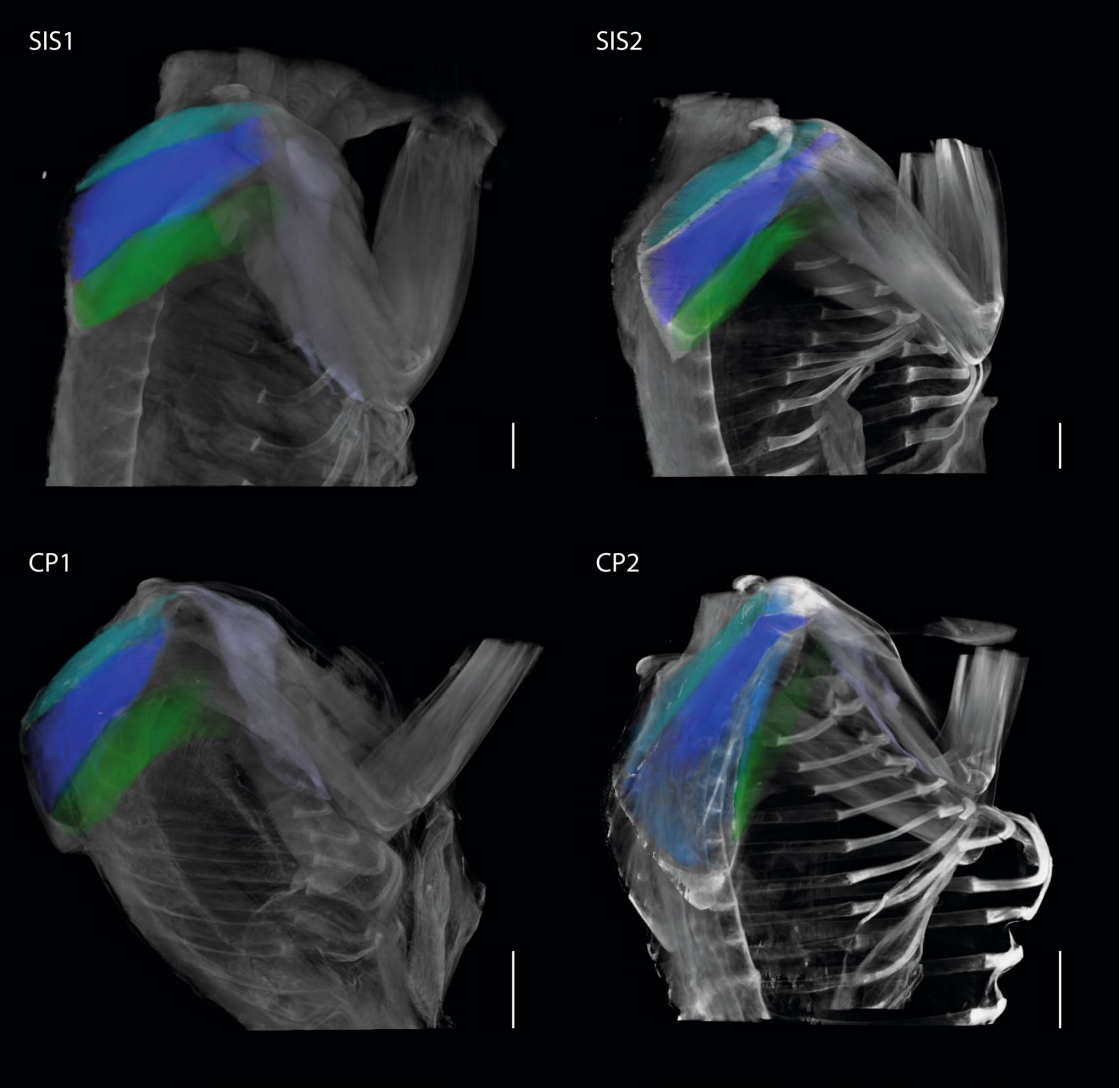
Lateral view of the shoulder muscles of *S. imperator* and *C. pygmaea.* SIS1 and SIS2: *S. imperator;* CP1 and CP2: *C. pygmaea*; supraspinatus: dark turquoise; infraspinatus: blue; teres major: dark green; pectoralis major: purple (due to damage not analyzed in this study). Scale bar = 10 mm

Absolute values for muscle volume for the four specimens analyzed can be found in Table 2. The relative volume of the triceps brachii, the brachialis, the deltoideus, and the supraspinatus is approximately twice as large in CP1 as in CP2 (Figs. 7, 8, 9). Similarly, the biceps brachii and the teres major are four times larger, and the subscapularis is one and a half times larger in CP1 than in CP2. The relative volume for all muscles considered is one and a half times larger in SIS1 than in SIS2. The relative muscle volume of *S. imperator* is larger than that of *C. pygmaea*.

**Table 2:** Muscle volume in mm^3^ (MV) and in relation to the average body mass of the corresponding species (MV_rel_)

Muscle		SIS1	SIS2	CP1	CP2*
Biceps brachii	MV	701.89	370.89	158.95	45.08
	MV_rel_	1.48	0.78	1.06	0.30
Triceps brachii	MV	2430.11	1415.65	329.64	171.85
	MV_rel_	5.12	2.98	2.20	1.15
Brachialis	MV	318.53	204.82	48.03	31.84
	MV_rel_	0.67	0.43	0.32	0.21
Deltoideus	MV	759.91	454.55	66.27	34.69
	MV_rel_	1.60	0.96	0.44	0.23
Teres major	MV	662.91	427.26	133.04	27.94
	MV_rel_	1.40	0.90	0.89	0.19
Supraspinatus	MV	583.04	378.72	89.80	44.31
	MV_rel_	1.23	0.80	0.60	0.30
Infraspinatus	MV	614.10	365.46	118.25	41.33
	MV_rel_	1.29	0.77	0.79	0.28
Subscapularis	MV	975.73	533.47	153.70	117.94
	MV_rel_	2.05	1.12	1.02	0.79

*Atrophied specimen marked with an asterisk

### Muscle length

Absolute values for muscle length for the four specimens analyzed can be found in Table 3. The two specimens of each species in our limited dataset did not exhibit pronounced differences. The specimens representing the same species did not show distinct differences in relative muscle length (Fig. 7). The relative muscle length did not show pronounced differences between the species.

**Table 3:** Muscle length in mm (ML) and in relation to the average body mass^0.33^ of the corresponding species (ML_rel_)

Muscle		SIS1	SIS2	CP1	CP2*
Biceps brachii	ML	45.9	37.6	31.0	29.5
	ML_rel_	5.89	4.82	5.84	5.55
Triceps brachii	ML	43.9	39.2	29.3	29.2
	ML_rel_	5.63	5.02	5.51	5.49
Brachialis	ML	37.1	34.8	24.1	26.2
	ML_rel_	4.75	4.45	4.54	4.93
Deltoideus	ML	24.0	20.9	19.0	18.9
	ML_rel_	3.08	2.68	3.58	3.55
Teres major	ML	34.4	32.9	19.0	23.8
	ML_rel_	4.41	4.21	3.57	4.48
Supraspinatus	ML	34.3	29.9	18.7	20.4
	ML_rel_	4.39	3.83	3.52	3.84
Infraspinatus	ML	35.4	35.1	20.0	22.8
	ML_rel_	4.54	4.50	3.76	4.29
Subscapularis	ML	30.7	26.9	18.8	21.1
	ML_rel_	3.94	3.44	3.54	3.97

*Atrophied specimen marked with an asterisk

### Anatomical cross-sectional area

Absolute values for ACSA for the four specimens analyzed can be found in Table 4. The relative ACSA of all muscles considered is approximately twice as large in CP1 as in CP2. The relative ACSA for biceps brachii, triceps brachii, brachialis, deltoideus, teres major, supraspinatus, infraspinatus, and subscapularis is slightly larger in SIS1 than in SIS2.

**Table 4:** ACSA of muscles in mm^2^ (ACSA) and in relation to the average body mass^0.66^ of the corresponding species (ACSA_rel_)

Muscle		SIS1	SIS2	CP1	CP2*
Biceps brachii	ACSA	15.28	9.87	5.13	1.53
	ACSA_rel_	0.25	0.16	0.18	0.05
Triceps brachii	ACSA	55.33	36.12	11.26	5.89
	ACSA_rel_	0.91	0.59	0.40	0.21
Brachialis	ACSA	8.59	5.89	1.99	1.22
	ACSA_rel_	0.14	0.10	0.07	0.04
Deltoideus	ACSA	31.61	21.71	3.48	1.48
	ACSA_rel_	0.52	0.36	0.12	0.07
Teres major	ACSA	19.26	13.00	7.01	1.17
	ACSA_rel_	0.32	0.21	0.25	0.04
Supraspinatus	ACSA	17.02	12.66	4.80	2.17
	ACSA_rel_	0.28	0.21	0.17	0.08
Infraspinatus	ACSA	17.34	10.40	5.92	1.82
	ACSA_rel_	0.28	0.17	0.21	0.06
Subscapularis	ACSA	31.77	19.86	8.17	5.59
	ACSA_rel_	0.52	0.33	0.29	0.20

*Atrophied specimen marked with an asterisk

The relative ACSA for biceps brachii, brachialis, teres major, supraspinatus, infraspinatus, and subscapularis is three times larger in *S. imperator* than in *C. pygmaea* (Fig. 7). The relative ACSA for triceps brachii is twice as large in *C. pygmaea* as in *S. imperator*. For deltoideus, the relative ACSA is three times larger in *S. imperator* than in *C. pygmaea*.

### Osteological muscle moment arms

SIS1 has a slightly larger relative distal moment arm than SIS2 for biceps brachii, triceps brachii, and deltoideus (Table 5). For brachialis, teres major, infraspinatus, and subscapularis, the relative distal moment arm of SIS1 is smaller in comparison to SIS2. The relative distal moment arm of supraspinatus is the same in both individuals. CP1 has a slightly smaller distal relative moment arm for biceps brachii, triceps brachii, brachialis, supraspinatus, infraspinatus, and subscapularis than CP2. For deltoideus and teres major, CP1 has a slightly larger relative moment arm than CP2. The relative proximal moment arm for biceps brachii caput longum and caput breve and for teres major is slightly larger in SIS2 than in SIS1. For the other muscles examined in this study the relative proximal moment arm is approximately the same in both specimens of *S. imperator*. The relative proximal moment arm for biceps brachii caput longum and caput breve, triceps brachii caput longum, deltoideus pars spinalis, teres major, and supraspinatus is slightly larger in CP1 than in CP2. For the other muscles, the relative proximal moment arm is approximately the same in both specimens of *C. pygmaea*.

**Table 5:** Osteological muscle moment arms in mm (MA) and in relation to the length of the connecting skeletal element (MA_rel_) for the distal (dMA) and the proximal (pMA) moment arm

Muscle		SIS1	SIS2	CP1	CP2*
Distal osteological muscle moment arms (dMA)					
Biceps brachii to radius	dMA	9.97	8.86	5.42	6.86
	dMA_rel_	0.23	0.22	0.17	0.20
Triceps brachii to ulna	dMA	6.19	5.61	3.45	3.93
	dMA_rel_	0.12	0.11	0.10	0.10
Brachialis to ulna	dMA	6.63	6.60	4.41	5.15
	dMA_rel_	0.13	0.13	0.12	0.13
Deltoideus to humerus	dMA	14.14	11.77	9.80	9.70
	dMA_rel_	0.28	0.26	0.28	0.26
Teres major to humerus	dMA	13.90	12.84	9.84	9.90
	dMA_rel_	0.27	0.28	0.28	0.26
Supraspinatus to humerus	dMA	4.43	3.89	2.78	3.22
	dMA_rel_	0.09	0.08	0.08	0.08
Infraspinatus to humerus	dMA	4.70	4.56	2.57	3.02
	dMA_rel_	0.09	0.10	0.07	0.08
Subscapularis to humerus	dMA	5.80	5.79	3.06	3.89
	dMA_rel_	0.11	0.13	0.09	0.10
Proximal osteological muscle moment arm (pMA)					
Biceps brachii caput longum to scapula	pMA	48.75	43.39	32.03	35.20
	pMA_rel_	1.60	1.72	1.74	1.48
Biceps brachii caput breve to scapula	pMA	47.01	42.09	31.32	35.55
	pMA_rel_	1.55	1.67	1.70	1.49
Triceps brachii caput longum to scapula	pMA	46.68	41.79	33.49	35.20
	pMA_rel_	1.76	1.77	1.81	1.70
Triceps brachii caput mediale to humerus	pMA	21.55	19.82	14.19	16.54
	pMA_rel_	0.42	0.43	0.41	0.44
Triceps brachii caput laterale to humerus	pMA	28.73	26.65	21.44	22.64
	pMA_rel_	0.56	0.58	0.61	0.60
Brachialis to humerus	pMA	26.69	24.44	13.11	15.60
	pMA_rel_	0.52	0.53	0.37	0.41
Deltoideus to clavicula	pMA	8.46	8.51	5.35	6.07
	pMA_rel_	0.32	0.35	0.32	0.33
Deltoideus to acromium	pMA	4.14	3.56	1.14	1.69
	pMA_rel_	0.15	0.14	0.06	0.08
Deltoideus to spina	pMA	18.85	16.70	6.35	6.41
	pMA_rel_	0.68	0.68	0.36	0.31
Teres major to scapula	pMA	32.59	30.03	21.10	21.07
	pMA_rel_	1.18	1.22	1.14	1.07
Supraspinatus to scapula	pMA	15.79	14.88	11.31	12.22
	pMA_rel_	0.57	0.61	0.64	0.59
Infraspinatus to scapula	pMA	21.38	17.64	14.13	15.71
	pMA_rel_	0.77	0.72	0.80	0.76
Subscapularis to scapula	pMA	20.13	17.23	13.44	15.20
	pMA_rel_	0.73	0.70	0.76	0.73

*Atrophied specimen marked with an asterisk

The relative distal moment arm is slightly larger in *S. imperator* for biceps brachii, triceps brachii, infraspinatus, and subscapularis in comparison to *C. pygmaea*. For brachialis, deltoideus, teres major, and supraspinatus the specimens do not exhibit pronounced differences. The relative proximal moment arm is larger in *S. imperator* for deltoideus pars acromialis and pars spinalis in comparison to *C. pygmaea*. For the other muscles, the relative proximal moment arm is approximately the same in both species.

## Discussion

Through a comparison of two species of callitrichid primate species faced with differing functional demands, the aim of this study was to provide incipient comparative data for the investigation of the relationship between the properties of the shoulder and brachial muscles, the muscle moment arms, and locomotor ecology. Contrast-enhanced µCT was used to visualize the muscles and to quantify functionally relevant parameters of the musculoskeletal system in a limited sample of primate specimens.

### Gross morphology

Functional specialization, such as a specialization to a specific locomotor mode like HL or TTL studied here, could result in changed positions of muscle attachment sites, which will alter potential muscle excursion (e.g., Lautenschlager 2015; Regnault et al. 2020) and muscle moment arms (e.g., Michilsens et al. 2009; Regnault et al. 2017). Overall, the muscle attachment sites are similar for *S. imperator* and *C. pygmaea*. Interspecific differences are observed in teres major and deltoideus, which leads to differences in moment arms for these muscles.

In relation to the species’ average body mass, *S. imperator* is characterized by very large muscle volumes. This is consistent with the assumption that *S. imperator* has comparatively larger and more powerful muscles due to its locomotor behavior (Leischner et al. 2018; Marchi et al. 2018). In *S. imperator* the triceps brachii has a relative volume twice as large as that of *C. pygmaea*, suggesting that in HL this muscle has a more significant role than in TTL. This muscle plays a crucial role as an anti-gravity muscle and mainly counteracts gravity-induced limb flexion in pronograde postures (Jacobs et al. 1993; Fischer and Blickhan 2006). It can also be expected to contribute to the dissipation of impact forces by becoming stretched after landing when the limb is flexed.

Of all the muscles examined, the greatest interspecific difference can be observed in the deltoideus and the teres major. For these muscles the relative muscle volume of *S. imperator* is four times larger than in *C. pygmaea*. The teres major plays a crucial role in climbing and serves as a humerus retractor (Argot 2001; Larson and Stern 1986; Toledo 2013). Relatively, *S. imperator* seems to invest more into this muscle than *C. pygmaea*. A strong teres major, which is important for humeral retraction, seems to be beneficial for horizontal leaping. The lower relative muscle volume in *C. pygmaea* potentially is linked to the TTL behavior, since an increase in body weight, which would be associated with a larger muscle volume, impairs locomotor capabilities on vertical surfaces following the argumentation of Karantanis (2010), as it becomes more difficult to counteract gravity in a vertical clinging position the heavier an animal gets. Also, the larger relative muscle volume in *S. imperator* may be related to the larger role of the forelimbs for the generation of propulsion during quadrupedal locomotion on moderately inclined surfaces, which has been demonstrated for similar sized cotton-top tamarins (Hesse et al. 2015). More comparative data is needed to assess these trends quantitatively.

### Muscle architecture

The ACSA is the ratio of the muscle volume to the muscle’s length. A relatively (to body mass) large ACSA is an indicator of muscles that can generate a lot of force. The relative ACSA of the muscles is larger in *S. imperator* than in *C. pygmaea* (Fig. 7). The biggest differences can be observed for triceps brachii and deltoideus, which are forelimb extensors and important on horizontal or declining supports, e.g., head-first postures, as well as during jumping and to dissipate energy with the forelimbs during landing, which appears to be more important for horizontal leapers. Muscles with a small ACSA (not taking into account the confounding factor of potential differences in fascicle lengths) also reduce the mass of the front limb and thereby suggest the capability for rapid joint rotation during powerful movements that might be advantageous for certain locomotor tasks, such as vertical climbing and leaping.

The relative muscle lengths did not differ between the species analyzed in this study, suggesting that muscle length is not affected by preferred locomotor behavior.

### Osteological muscle moment arms

Moment arms together with force generated by a muscle determine the torque acting at a joint (Channon et al. 2010). The quantitative description of moment arms is essential to understanding the function of the musculoskeletal system (Murray et al. 2002; Ackland et al. 2008; Channon et al 2010). The larger the moment arm, the greater the torque that can be generated, but the lower the speed of joint movement (Lieber and Fridén 2001; Channon et al. 2010). This very general pattern is also dependent on contraction speed, which is determined by fascicle length but not captured in ACSA (e.g., Lieber and Fridén 2001; Allen et al. 2010). The forelimb muscles of sloths are an extreme example where moment arms are maximized and locomotor speed is limited (Fujiwara et al. 2011; Nyakatura and Fischer 2011).

The only difference in relative distal moment arm is observed for biceps brachii and triceps brachii. The relative distal moment arm is slightly larger in *S. imperator* than in *C. pygmaea*. For relative proximal moment arm, the only difference is observed for deltoideus pars acromialis and pars spinalis. The relative moment arm for these muscles is twice as large in *S. imperator* as in *C. pygmaea*. These differences in moment arms suggest a larger importance of counterbalancing arm flexion during landing for these muscles.

The relative distal moment arms of triceps brachii, brachialis, supraspinatus, infraspinatus, and subscapularis and the relative proximal moment arms of triceps brachii caput mediale, triceps brachii caput laterale, deltoideus pars clavicularis, deltoideus pars acromialis, deltoideus pars spinalis, supraspinatus, infraspinatus, and subscapularis are small in both species examined compared to the rest of the examined muscles, which suggests that these muscles might have a stabilizing function in the shoulder muscles (Ackland et al. 2008). In addition, a small moment arm increases a muscle's working range (Channon et al. 2010). The large relative distal moment arms of biceps brachii, deltoideus, and teres major and the relative proximal of biceps brachii, triceps brachii caput longum, and teres major in both species characterize these muscles as prime movers and suggest that they are not suitable for rapid movements during jumping, but to generate great forces. For example, Payne et al. (2006) have shown that instantaneous muscle moment arms increase with increasing joint flexion, a finding which may be understood in the context of employment of more crouched and compliant postures during arboreal locomotion (Schmitt 1999). A predominant role in stability in fact may not be reflected in the lengths of moment arms, but for example in fiber-type composition which was not assessed here.

### Limitations

A limitation of this study may be the small number of available individuals. This limits the expressiveness of intraspecific differences, since musculoskeletal differences between two individuals could theoretically be traced back to illnesses or an age-related decrease in muscles. The age-related decrease in musculature and the associated decrease in muscle strength was observed in all mammals that were examined for age-related changes (Faulkner et al. 2007, 2008). The specimens did not show obvious signs of disease or illness. Nevertheless, one specimen appeared to be atrophied (CP2). We marked the specimen with an asterisk in all graphs and tables and considered potentially flawed muscle volume and ACSA data for this specimen in the discussion.

In both wild and captive animals, age is usually accompanied by a decrease in grip strength (Hamalainen et al. 2015). The analyzed subjects stem from a captive breeding group, which can have an influence on the pattern of food intake and on locomotor behavior related to predator avoidance. The absence of such ecological pressures allows older individuals to maintain their health longer than would be possible in the wild. Accordingly, animals kept in captivity often develop a locomotor repertoire that differs from that of their wild counterparts (Crompton et al. 2003). Finally, we cannot rule out that the muscle tissue, due to the long storage time in alcohol and the treatment with contrast media, has shrunk (Cutts 1988; Vickerton et al. 2013; Buytaert et al. 2014). Signs of this include that the volumes of the bones of CP2 are larger than in CP1, but the volumes of the muscles are smaller. In addition, a few superficial muscles of CP2 and SIS2 appeared to be damaged by freezer burn (Table. 1). We acknowledge that differences in anatomy cannot be attributed to functional requirements alone, but also other factors like age, and training condition might have an influence. Sexual dimorphism might also be a factor but is not common in small platyrrhine primates and therefore not taken into account in this study (e.g., Kappeler 1990; Ford 1994). Nevertheless, we believe that the general patterns that we describe here are reflective of the species’ adaptiveness to their differing ecology, and the comparison of such closely related species may thus provide us with tentative insight into the functional adaption of these primates. The advent of algorithms that allow more automated segmentation (e.g., Dickinson et al. 2018; Nyakatura et al. 2019) and availability of even higher-resolution µCT imaging could alleviate the time-consuming manual segmentation of CT images and could provide an avenue towards the comparative analysis of larger and more inclusive datasets.

## Conclusion

We examined the functional relationship between the properties of the musculoskeletal system of the shoulder and the locomotor behavior in the vertical clinger and leaper *C. pygmaea* and in *S. imperator*, a species that prefers horizontal locomotion*.* Despite the acknowledged limitations, the following conclusions can tentatively be drawn. Compared to *S. imperator*, *C. pygmaea* has muscles that are relatively small in volume. A low muscle mass might be advantageous for the long episodes of clinging to tree trunks involved in exudate feeding. The shoulder muscles of *S. imperator*, however, due to its relatively larger volume, are suited for the generation of larger forces. This might be beneficial for leaping from terminal branches that also involve the generation of thrust in the forelimbs. Although the moment arms do not differ very much between the two species, our results demonstrate that the preferred locomotor mode is reflected in the properties of the musculoskeletal system.

Since thrust for the jump is largely generated by the muscles of the hind limbs (Marchi et al. 2018), studies of the hind limb muscles and the length and orientation of the muscle fibers would be of great interest and could provide information about the functional differentiation of fore- and hindlimbs in arboreal locomotion. An examination of additional callitrichid primate species could yield improved insight into the morphofunctional adaptations within the clade.
